# Epistatic Interaction of *ERAP1* and HLA-B*51 in Iranian Patients with Behçet’s Disease

**DOI:** 10.1038/s41598-018-35700-0

**Published:** 2018-12-04

**Authors:** Mahdi Mahmoudi, Amir Ashraf-Ganjouei, Ali Javinani, Farhad Shahram, Akira Meguro, Nobuhisa Mizuki, Nooshin Ahmadzadeh, Saeideh Jafarinejad-Farsangi, Shayan Mostafaei, Hoda Kavosi, Seyedeh Tahereh Faezi, Maassoumeh Akhlaghi, Fereydoun Davatchi

**Affiliations:** 1 Rheumatology Research Center, Tehran University of Medical Sciences, Tehran, Iran; 2 Department of Ophthalmology and Visual Science, Yokohama City University Graduate School of Medicine, Yokohama, Japan; 3 Physiology Research Center, Institute of Basic and Clinical Physiology Sciences, Kerman University of Medical Sciences, Kerman, Iran; 4 Department of Community Medicine, Faculty of Medicine, Kermanshah University of Medical Sciences, Kermanshah, Iran

## Abstract

Behçet’s Disease (BD) pathogenesis remains unclear, but some genetic loci and environmental factors are proposed to play a role. Here, we investigate the association of the endoplasmic reticulum aminopeptidase-1 (*ERAP1*) gene variants and HLA-B*51 with BD susceptibility and clinical manifestations in Iranian patients. In the study, 748 BD patients and 776 healthy individuals were included. The MGB-TaqMan Allelic Discrimination method was used to genotype 10 common missense single nucleotide polymorphisms (SNPs) and one intronic SNP in the *ERAP1* gene region. We found no significant association between the 11 SNPs and BD in allelic and genotypic association tests. However, rs30187 showed the strongest association with BD in the recessive genotype model of the risk T allele in HLA-B*51 carriers. Although this became insignificant after correcting for multiple comparisons, the homozygous rs30187 risk allele genotype (TT) increased disease susceptibility in HLA-B*51 carriers in epistasis analysis, and the rs30187 TT recessive genotype showed a significant association with risk of cardiac involvement in the all patients and articular involvements in HLA-B*51 positive patients. Our findings suggest that gene-gene interactions between HLA-B*51 and *ERAP1* variants is important for BD development, however, *ERAP1* variants which interact with HLA-B*51 may differ among disease phenotypes or populations.

## Introduction

Behçet’s Disease (BD) is a rare multi-organ vasculitic disorder, first described by Hippocrates in the *Epidemion*^1^. In addition to ophthalmological and dermatological manifestations, oral and genital aphthosis are the main criteria for BD diagnosis^2,3^. Although BD is not prevalent in America (5.2 per 100,000) or Europe (2.4 per 100,000), its management continues to be a challenge for Asian rheumatologists due to the notably higher prevalence of BD in Silk Road countries including Iran, in which the point prevalence is 80 per 100,000^4,5^.

A major hypothesis for the etiology of autoimmune disorders is an exaggerated immune system response stimulated by endogenous and exogenous factors in a susceptible genetic background^6^. Previous work has attempted to uncover the genetic mechanisms involved in BD. Single nucleotide polymorphism (SNP) analyses indicated that a variety of genes may play a critical role in such exaggerated responses. Polymorphisms in genes including interleukin-2 (*IL2*), *IL4*, *IL6*, tumor necrosis factor-α (*TNFα*), and transforming growth factor-β (*TGFβ*) have been shown to have a probable role in BD pathogenesis^7,8^.

Endoplasmic reticulum aminopeptidase-1 (ERAP1) is a multifunctional zinc-metallopeptidase belonging to the M1 family of amino peptidases, with several immunologically important functions. The *ERAP1* gene is located on chromosome 5q15 and has a length of 53 kbp^9^. ERAP1 loads endoplasmic peptides on major histocompatibility complex (MHC) class I molecules to be presented to the immune system. These peptides are produced from both self and non-self-proteins, which are then degraded in the cytoplasm by a proteasome complex. Therefore, it is highly probable that different *ERAP1* gene polymorphisms can lead to different types of peptide loading and hyperactivation of the immune system^9^. In addition to antigen presenting, ERAP1 has a critical role in modulating inflammatory processes by cleaving and shedding the extracellular domain of pro-inflammatory molecule receptors, causing their inactivation. For instance, direct contact between ERAP1 and the TNF receptor 1 (TNFR1) extracellular domain *in vitro* results in the release of the 7–34 kDa soluble TNFR1 domain, culminating in TNFR1 inactivation and inhibition of the TNF-α mediated inflammatory pathways^10^. This event occurs similarly for IL-6 receptor-α (IL-6Rα) and IL-1 receptor II (IL-1RII)^11^. It is probable that different polymorphisms lead to more or less activated ERAP1, causing hyper or hypo-activation of various inflammatory processes.

Kirino *et al*. reported that the missense coding *ERAP1* SNP Arg725Gln (rs17482078) significantly affected BD risk in HLA-B*51 carriers in a Turkish population, suggesting a gene-gene interaction between *ERAP1* rs17482078 and HLA-B*51 in BD^12^ like that found between *ERAP1* variants and the disease-associated *HLA* alleles observed in psoriasis and ankylosing spondylitis (AS)^13,14^. Replication studies also suggested possible gene-gene interactions between *ERAP1* variants and HLA-B*51 in BD in Spanish and Iranian populations^15,16^, although the strengths of the interactions vary among these studies. Recently, Takeuchi *et al*. analyzed 10 missense *ERAP1* SNPs using the same Turkish cohort as Kirino *et al*. and found that one *ERAP1* haplotype (named Hap10) with five non-ancestral amino acids was recessively associated with BD in HLA-B*51 carriers^17^. They also reported that three of the five non-ancestral amino acids (Met349Val (rs2287987), Asp575Asn (rs10050860), and Arg725Gln (rs17482078)) are good tags for Hap10^17^.

In this study, we investigate the association between *ERAP1* SNPs and BD in an Iranian population. We also assess the gene-gene interaction between *ERAP1* SNPs and HLA-B*51 and the association of *ERAP1* SNPs with clinical manifestations of BD.

## Material and Method

### Study participants

In this study, 748 Iranian unrelated BD patients (less than 16 years old were excluded) who had been referred to the outpatient Behçet’s unit, Rheumatology Research Center, Shariati Hospital, Tehran, Iran, were selected by simple random sampling approach. All diagnoses were confirmed by the International Criteria for Behçet’s Disease (ICBD)^5,16^. The control group consisted of 776 age-, sex-, and ethnicity- matched healthy individuals, with no family history or clinical manifestation of any type of rheumatic or other autoimmune disorders. Of the 748 BD patients who participated in the study, 448 were men (59.9%) and 300 were women (40.1%) with a mean age of 40.26 ± 10.88 SD, ranging from 16 to 73 years. The control group consisted of 476 men (61.3%) and 300 women (38.7%) with a mean age of 38.88 ± 11.54 SD ranging from 16 to 75 years. The study protocol was approved by the ethical committee of Tehran University of Medical Sciences (Ethical Committee ID. 91-04-41-19380-296371) and written informed consent was obtained from all participants. For case and control subjects under the age of 18 years, informed consent was obtained from a parent and/or legal guardian. Alternately, all experiments were performed in accordance with relevant guidelines and regulations provided by the university.

### DNA preparation and SNP genotyping

A peripheral blood sample was collected from all participants into EDTA-anticoagulated tubes using venipuncture. Genomic DNA was extracted using the standard phenol/chloroform method^18^ and the extracted DNA samples were stored at −20 °C. Approximately 20 ng of the genomic DNA in each sample was used for genotyping.

We evaluated 10 common missense SNPs with minor allele frequency >1% identified in the EUR superpopulation of the 1000 Genomes Project, which were assessed in a previous study (Table 1)^17^. We also selected one intronic SNP (rs1065407) because of a study reporting its association with BD in a Chinese population without assessing the interaction between rs1065407 and HLA-B*51 (Table 1)^19^. SNP genotyping was performed using the MGB-TaqMan Allelic Discrimination method (Applied Biosystems, Foster City, CA, USA). Amplification was performed in 10 μl reaction volumes, containing 5 μl of the TaqMan genotyping master mix, 0.25 μl of TaqMan genotyping assay mix, 0.25 μl of distilled water, and 4.5 μl of genomic DNA. Patient and control samples were genotyped using the StepOnePlus Real-Time PCR System (Applied Biosystems) according to the manufacturer’s protocols. The allelic call was performed by the analysis of allelic discrimination plots, using SDS v.1.4 software (Applied Biosystems).

**Table 1:** Allelic association tests of 11 *ERAP1* SNPs.

SNP	Position on Chr. 5 (GRCh38)	Alleles (1 > 2)	Amino acid changes	ALL				HLA-B*51+			
				Minor allele freq., %		*P*	OR (95% CI)	Minor allele freq., %		*P*	OR (95% CI)
				Cases (N = 748)	Controls (N = 776)			Cases (N = 445)	Controls (N = 184)		
rs1065407	96,776,379	T > G	Intronic	36.6	32.5	0.018	1.20 (1.03–1.39)	38.4	34.9	0.25	1.16 (0.90–1.50)
rs27044	96,783,148	C > G	Glu730Gln	28.5	29.1	0.74	0.97 (0.83–1.14)	27.8	28.1	0.90	0.98 (0.75–1.29)
rs17482078	96,783,162	C > T	Arg725Gln	12.6	10.3	0.052	1.25 (1.00–1.56)	13.6	12.0	0.43	1.16 (0.80–1.68)
rs10050860	96,786,506	C > T	Asp575Asn	12.5	10.1	0.039	1.27 (1.01–1.59)	13.5	12.0	0.48	1.14 (0.79–1.65)
rs30187	96,788,627	C > T	Arg528Lys	40.1	39.7	0.82	1.02 (0.88–1.18)	39.7	36.3	0.27	1.15 (0.90–1.48)
rs2287987	96,793,832	T > C	Met349Val	12.5	10.2	0.040	1.27 (1.01–1.59)	13.5	12.3	0.57	1.11 (0.77–1.61)
rs27895	96,793,840	C > T	Gly346Asp	9.8	9.9	0.98	1.00 (0.79–1.26)	10.4	9.0	0.45	1.18 (0.77–1.78)
rs26618	96,795,133	T > C	Ile276Met	20.1	22.9	0.059	0.85 (0.71–1.01)	18.8	24.5	0.022	0.71 (0.53–0.95)
rs26653	96,803,547	G > C	Pro127Arg	40.2	39.7	0.75	1.02 (0.89–1.18)	40.7	35.2	0.070	1.26 (0.98–1.63)
rs3734016	96,803,761	C > T	Glu56Lys	1.9	2.4	0.40	0.81 (0.50–1.32)	2.1	1.6	0.57	1.31 (0.52–3.30)
rs72773968	96,803,892	G > A	Thr12Ile	9.8	9.9	0.88	0.98 (0.77–1.25)	9.3	10.1	0.66	0.91 (0.61–1.37)

1, major allele; 2, minor allele; OR, odds ratio; CI, confidence interval.

*P* < 0.00455 (0.05/11 SNPs) was considered significant after Bonferroni correction.

### Statistical analysis

Allelic and genotypic associations of the *ERAP1* SNPs with BD were evaluated by Pearson’s χ^2^ test using SNP & Variation Suite software version 8.6.0 (Golden Helix, Bozeman, MT, USA). The genotype distributions of SNPs were tested for deviation from Hardy-Weinberg equilibrium (HWE) in control group. Two risk factor analyses (HLA-B*51 and the T allele of rs30187) were evaluated by 2 × 2 contingency table ORs comparing the frequency in cases with controls of the single-risk factor or two-risk factor groups, relative to the frequency of individuals with neither risk factor. *P* values were corrected for multiple comparisons using the Bonferroni correction, in which where 0.05 was divided by the number of comparisons to assess the adjusted significance level. The pairwise linkage disequilibrium was calculated using Haploview version 4.2 software (Broad Institute, Cambridge, MA, USA)^20^.

## Results

The distribution of the genotypes for all *ERAP1* SNPs in healthy control group did not demonstrate any significant deviation from the HWE. Table 1 shows the allelic association results for the 11 tested SNPs in all participants and HLA-B*51 positive participants alone. Three SNPs (rs1065407, rs10050860, and rs2287987) had a nominally significant association with BD in all subjects, and one SNP (rs26618) had a nominally significant association with HLA-B*51 positive subjects (*P* < 0.05). However, these results were not significant after Bonferroni correction for multiple comparisons. There was no evidence for a gene-gene interaction between the *ERAP1* SNPs and HLA-B*51 in the allelic association analyses.

**Figure 1:**
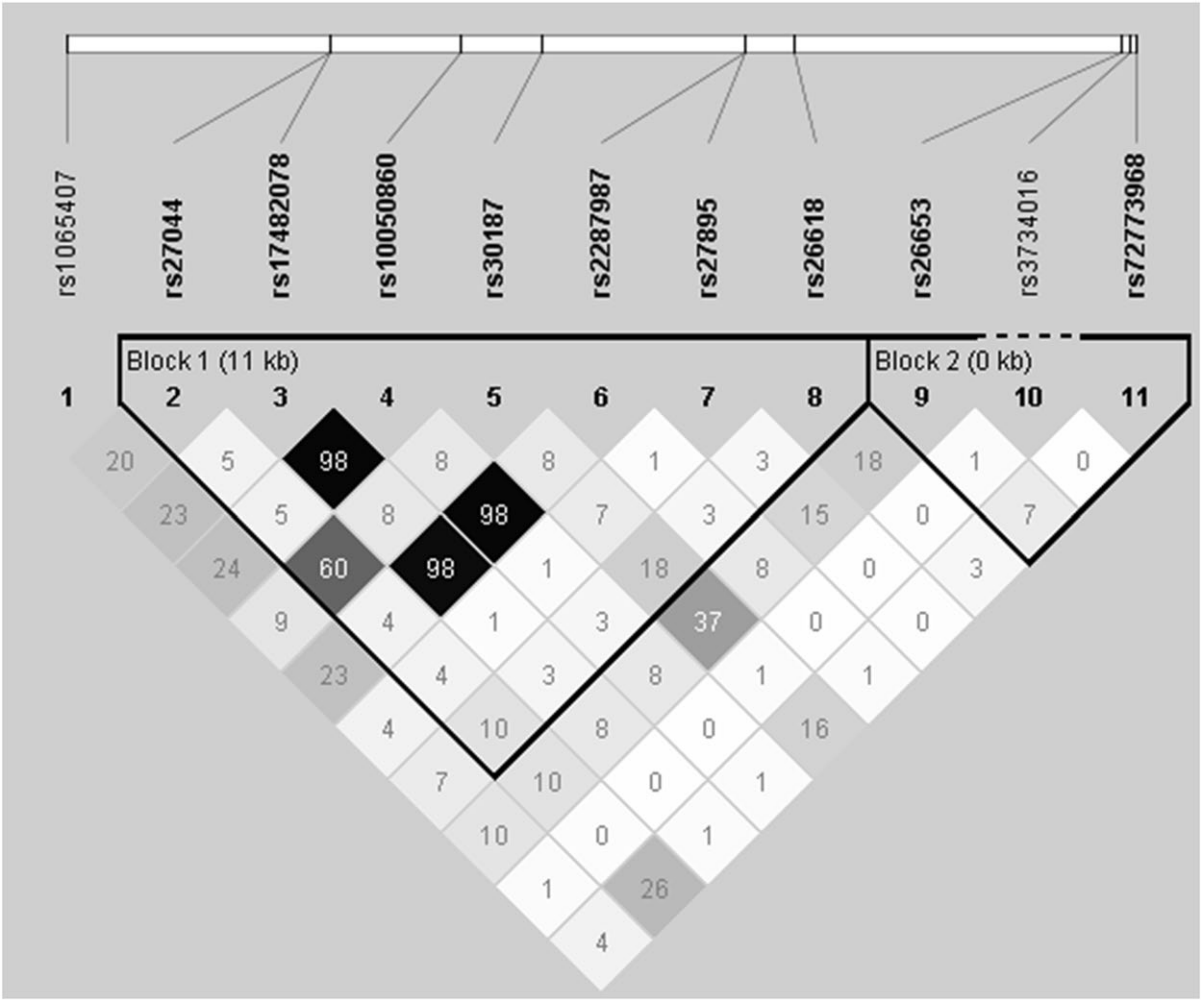
Linkage disequilibrium plot of 11 *ERAP1* SNPs among 1,524 study participants. The *r*^2^ value corresponding to each SNP pair is expressed as a percentage and shown within the respective square. Shading represents the magnitude and significance of pairwise LD, with the white to black gradient reflecting lower to higher *r*^2^ values.

Table 2 summarizes the genotypic association results for the 11 SNPs, calculated for the dominant and recessive inheritance models. The strongest association signal was observed for rs30187 in the recessive model of the risk T allele (Arg528Lys) in HLA-B*51 carriers (*P* = 0.0077, OR = 2.07), which did not show any association with BD in the recessive model in the whole study population (*P* = 0.15, OR = 1.22). This finding suggests an epistatic interaction between the rs30187 TT genotype and HLA-B*51, but the association did not reach statistical significance after applying the Bonferroni correction. rs26653 was in moderate linkage disequilibrium (LD) with rs30187 (*r*^2^ = 0.37) (Fig. 1) and also showed a significant association in the recessive model of its risk C allele (Pro127Arg) in HLA-B*51 carriers (OR = 2.00) but not in the whole population. The association also failed to reach statistical significance after Bonferroni correction. The non-ancestral alleles (Met349Val, Asp575Asn, and Arg725Gln) of the three SNPs (rs2287987, rs10050860, and rs17482078, respectively) previously reported as good tags for Hap10 showed a significant association before Bonferroni correction with an OR > 2.5 in the recessive model in the whole population, while the association was not significant with an OR < 2.0 in the recessive model in the HLA-B*51 carriers, suggesting that they have no epistatic interaction with HLA-B*51 in our Iranian population. These three SNPs were not in LD with rs30187 (*r*^2^ = 0.08) (Fig. 1). No statistically significant results were detected after Bonferroni correction for the remaining six SNPs and no possible epistatic interaction with HLA-B*51 were observed.

**Table 2:** Genotypic association tests of 11 *ERAP1* SNPs.

SNP	Alleles (1 > 2)	Phenotype	Genotype ([2/2]/[1/2]/[1/1]) frequency, %		Dominant model ([2/2] + [1/2] vs. [1/1])		Recessive model ([2/2] vs. [1/2] + [1/1])	
			Cases	Controls	*P*	OR (95% CI)	*P*	OR (95% CI)
rs1065407	T > G	ALL	13.6/46.1/40.3	11.5/42.0/46.4	0.016	1.28 (1.05–1.57)	0.23	1.21 (0.89–1.64)
		HLA-B*51+	15.0/46.8/38.3	11.5/46.7/41.8	0.41	1.16 (0.81–1.64)	0.26	1.35 (0.80–2.28)
rs27044	C > G	ALL	7.0/43.2/49.9	5.3/47.6/47.1	0.28	0.90 (0.73–1.09)	0.18	1.34 (0.88–2.04)
		HLA-B*51+	7.6/40.4/52.0	4.9/46.4/48.6	0.44	0.87 (0.62–1.23)	0.23	1.59 (0.75–3.38)
rs17482078	C > T	ALL	2.8/19.5/77.7	1.0/18.6/80.4	0.19	1.18 (0.92–1.51)	0.011	2.77 (1.22–6.29)
		HLA-B*51+	4.2/18.8/77.0	2.2/19.6/78.3	0.73	1.07 (0.71–1.63)	0.21	1.99 (0.67–5.94)
rs10050860	C > T	ALL	2.7/19.7/77.7	1.0/18.2/80.8	0.14	1.21 (0.94–1.55)	0.017	2.63 (1.15–6.02)
		HLA-B*51+	4.0/19.0/77.0	2.2/19.7/78.1	0.76	1.07 (0.71–1.61)	0.25	1.87 (0.63–5.61)
rs30187	C > T	ALL	18.0/44.2/37.8	15.2/48.9/35.9	0.44	0.92 (0.75–1.13)	0.15	1.22 (0.93–1.60)
		HLA-B*51+	18.4/42.6/39.0	9.8/53.0/37.2	0.66	0.92 (0.65–1.32)	0.0077	2.07 (1.20–3.55)
rs2287987	T > C	ALL	2.6/19.9/77.5	1.0/18.2/80.7	0.13	1.21 (0.95–1.56)	0.024	2.52 (1.10–5.80)
		HLA-B*51+	3.9/19.3/76.9	2.2/20.2/77.6	0.84	1.04 (0.69–1.57)	0.29	1.79 (0.60–5.41)
rs27895	C > T	ALL	1.5/16.7/81.8	0.8/18.2/81.1	0.70	0.95 (0.73–1.23)	0.20	1.92 (0.70–5.21)
		HLA-B*51+	1.6/17.6/80.8	0.0/17.9/82.1	0.71	1.09 (0.70–1.69)	0.088	—
rs26618	T > C	ALL	4.4/31.4/64.2	5.3/35.3/59.4	0.056	0.82 (0.66–1.01)	0.43	0.83 (0.52–1.32)
		HLA-B*51+	3.8/29.9/66.3	5.4/38/56.5	0.020	0.66 (0.47–0.94)	0.35	0.69 (0.31–1.53)
rs26653	G > C	ALL	16.2/48.1/35.7	15.2/48.9/35.9	0.94	1.01 (0.82–1.24)	0.61	1.07 (0.82–1.42)
		HLA-B*51+	16.1/49.3/34.6	8.7/53.0/38.3	0.38	1.17 (0.82–1.67)	0.016	2.00 (1.13–3.54)
rs3734016	C > T	ALL	0.1/3.6/96.3	0.0/4.8/95.2	0.32	0.78 (0.47–1.28)	0.31	—
		HLA-B*51+	0.2/3.8/96.0	0.0/3.3/96.7	0.65	1.24 (0.48–3.18)	0.52	—
rs72773968	G > A	ALL	1.1/17.4/81.6	0.5/18.8/80.7	0.66	0.94 (0.73–1.22)	0.22	2.09 (0.63–6.96)
		HLA-B*51+	1.3/15.8/82.8	0.0/20.1/79.9	0.39	0.82 (0.53–1.28)	0.11	—

1, major allele; 2, minor allele; OR, odds ratio; CI, confidence interval.

*P* < 0.00455 (0.05/11 SNPs) was considered significant after Bonferroni correction.

The combinatory effect of HLA-B*51 and the homozygous risk allele genotype (TT) of rs30187 on disease susceptibility is shown in Table 3. HLA-B*51 negative/non-TT carriers were the reference. The TT genotype does not increase disease susceptibility risk in the absence of the HLA-B*51 gene (*P* = 0.78, OR = 1.05). HLA-B*51 in non-TT carriers of the rs30187 locus increased BD susceptibility risk with an OR of 4.47 (*P* = 4.01 × 10^−36^). The combination of HLA-B*51 positivity and homozygous TT rs30187 genotype further increased the risk of disease susceptibility with an OR of 9.22 (*P* = 4.64 × 10^−21^).

**Table 3:** Two risk factor analysis between HLA-B*51 and the T allele of rs30187.

HLA-B*51/homozygous for rs30187 T allele	Number of cases, n (%)	Number of controls, n (%)	*P* ^*^	OR (95% CI)
−/−	243 (32.8)	492 (63.5)	—	1.00 Reference
−/+	52 (7.0)	100 (12.9)	0.78	1.05 (0.73–1.52)
+/−	364 (49.1)	165 (21.3)	4.01 × 10^−36^	4.47 (3.51–5.68)
+/+	82 (11.1)	18 (2.3)	4.64 × 10^−21^	9.22 (5.41–15.71)

OR, odds ratio; CI, confidence interval.

^*^P < 0.0167 (0.05/3 groups with one or more risk factors) was considered significant after Bonferroni correction.

We also assessed the association of the rs30187 homozygous TT genotype with clinical manifestations in BD patients (Table 4). The T allele of rs30187 increased the risk of cardiac involvement under the recessive model regardless of HLA-B*51 status (OR = 11.14 in the whole population; OR = 9.17 in the HLA-B*51 carriers) and the risk was significant in the whole population (*P* = 0.00055) but not in the HLA-B*51 carriers alone (*P* = 0.063). The OR of the T allele under the recessive model was higher in the HLA-B*51 carriers than in the whole population for all of the clinical manifestations except for cardiac involvement, and the homozygous TT genotype was significantly associated with the risk of arthritis in HLA-B*51 positive patients (*P* = 0.0013, OR = 2.54). In addition, the homozygous TT genotype showed a higher risk for neurologic involvement (OR = 2.55) and epididymitis (OR = 3.18) than arthritis in the HLA-B*51 carriers, although the risks were not significant.

**Table 4:** Recessive effects of the T allele of rs30187 on clinical symptoms of Behçet’s Disease.

Phenotype	ALL			HLA-B*51+		
	Prevalence of phenotype, %	Recessive model (TT vs. CT + CC)		Prevalence of phenotype, %	Recessive model (TT vs. CT+CC)	
		*P*	OR (95% CI)		*P*	OR (95% CI)
Oral aphthosis	98.39	0.16	1.22 (0.93–1.60)	98.88	0.0064	2.10 (1.22–3.61)
Genital aphthosis	62.82	0.14	1.26 (0.93–1.71)	66.22	0.0093	2.10 (1.19–3.71)
Pseudofolliculitis	48.72	0.082	1.33 (0.96–1.85)	49.44	0.0049	2.29 (1.27–4.13)
Erythema nodosum	28.05	0.48	1.16 (0.77–1.74)	30.65	0.029	2.05 (1.07–3.93)
Ophthalmological involvement	66.58	0.89	0.98 (0.71–1.34)	66.00	0.057	1.74 (0.98–3.11)
Arthritis	51.41	0.033	1.41 (1.03–1.94)	51.01	0.0013	2.54 (1.42–4.53)
Vascular involvement	11.14	0.92	1.03 (0.55–1.93)	11.41	0.24	1.71 (0.69–4.19)
Neurologic involvement	4.83	0.49	1.34 (0.58–3.14)	5.15	0.088	2.55 (0.84–7.68)
Epididymitis	3.22	0.47	1.45 (0.53–4.01)	3.58	0.067	3.18 (0.88–11.57)
Gastrointestinal involvement	3.22	0.71	0.80 (0.23–2.71)	2.46	0.94	0.92 (0.11–7.58)
Cardiac involvement	0.81	0.00055	11.14 (2.02–61.49)	0.45	0.063	9.17 (0.55–152.90)
Positive pathergy test	45.10	0.085	1.34 (0.96–1.87)	52.35	0.010	2.13 (1.19–3.84)

OR, odds ratio; CI, confidence interval.

The frequencies of signs were calculated among patients. Ophthalmological manifestation consists of anterior uveitis, posterior uveitis and retinal vasculitis. Note that epididymitis was calculated in the male population of the patients. *P* < 0.00417 (0.05/12 phenotypes) was considered significant after Bonferroni correction.

## Discussion

We aimed to investigate the association between *ERAP1* SNPs and BD in an Iranian population. To the best of our knowledge, this is the first study to evaluate *ERAP1* SNPs in Iranian patients with BD. We found compelling evidence for an epistasis between HLA-B*51 and rs30187 T allele homozygotes (Arg528Lys) in the Iranian patients with BD.

Homozygous Met349Val (rs2287987), Asp575Asn (rs10050860), and/or Arg725Gln (rs17482078) have been previously reported to show an epistatic interaction for BD susceptibility with HLA-B*51. Previous studies in a Turkish population reported a gene-gene interaction for disease susceptibility between HLA-B*51 and homozygous Arg725Gln or Hap10, including five non-ancestral amino acids (Met349Val, Arg528Lys, Asp575Asn, Arg725Gln, and Gln730Glu)^12,17^. Conde-Jaldón *et al*. also found the strongest interaction between HLA-B*51 and homozygosity for a haplotype consisting of five non-ancestral amino acids in a Spanish population^15^. Together, these results suggest that interactions between Met349Val, Asp575Asn, and Arg725Gln and HLA-B*51 are important in BD. This is in contrast with the results of the current study, which found no evidence for an epistatic interaction between HLA-B*51 and these three non-ancestral amino acids. Only one study in a Spanish population suggested a gene-gene interaction between HLA-B*51 and homozygosity of the rs30187 T allele (Arg528Lys) when a haplotype consists of Arg528Lys and Glu730Gln (rs27044), however the strength of the interaction was less than that of the haplotype containing Met349Val, Asp575Asn, and Arg725Gln^15^.

There are at least two reasons explaining the disparity between our results and those reported in the previous studies. First, the disparity may be explained by the genetic diversity among ethnic groups in BD susceptibility, because differences in genetic backgrounds between groups could affect association levels of genetic factors for disease susceptibility. However, this explanation does not seem to be complete due to the close similarity in the genetic backgrounds of the Iranian and Turkish populations. Second, differences in the phenotypes of BD patients may lead to different results among studies. BD encompasses a wide variety of clinical manifestations and is not a single condition, therefore the clinical features of BD patients differ among studies. This study suggests that the rs30187 T allele (Arg528Lys) is recessively associated with the development of several clinical manifestations in BD, therefore, the association of Arg528Lys with BD may be affected by the disease phenotype.

Sousa *et al*. previously evaluated the association between *ERAP1* SNPs and BD in a Iranian population and reported marginal evidence for an interaction between HLA-B*51 and homozygous Asp575Asn (rs10050860) and Arg725Gln (rs17482078)^16^, which differs from our results. There are two major differences between our studies. First is the number of tested SNPs. They analyzed only rs10050860 and rs17482078 and therefore they could not comprehensively detect relationships between *ERAP1* SNPs such as rs30187 and BD. Second is the difference in the participants sampled. More than half of the BD patients and all of the controls used in this study are different from those included in Sousa *et al*., and the phenotypes of BD patients differ between the two studies. For example, the prevalence of arthritis is 51.41% in this study and 30.8% in Sousa *et al*., and the prevalence of vascular involvement is 11.14% in this study and 5.2% in Sousa *et al*. Phenotypic differences may lead to different association results for Asp575Asn and Arg725Gln with BD between the two Iranian cohorts.

The association of *ERAP1* rs30187 with clinical phenotypes is well investigated in AS, a type of arthritis affecting the joints in the spine. Wang *et al*. reported that the rs30187 T allele was significantly associated with syndesmophyte formation in AS patients in a Taiwanese population^21^. Nossent *et al*. also reported that CT haplotype of rs27044/rs30187 was associated with a reduced risk of extra-spinal manifestations, including uveitis in Caucasian HLA-B*27 positive patients with AS^22^. Individuals with the TT genotype of rs30187 in a Romanian population were approximately three times more susceptible to psoriatic arthritis, another type of spondyloarthritis with similar symptoms to AS^23^. In this study, the rs30187 T allele was recessively associated with arthritis in HLA-B*51 positive BD patients. These data suggest that the rs30187 T allele affects clinical phenotypes, especially arthritis, in BD as well as in AS. This relationship requires investigation in further studies.

Trimmed peptides loaded on the MHC class I proteins are significantly affected by ERAP1 activity and structure. Consequently, these trimmed peptides can trigger the immune system in different ways. Guasp *et al*. reported that an *ERAP1* variant with low activity trimmed peptides with low affinity for HLA-B51 and favored NK cell cytotoxicity^24^. Molecular modeling of ERAP1 indicates that rs30187 (Arg528Lys) is located next to the entrance of the substrate pocket. Lysine (Lys) replacement with Arginine (Arg) or any other amino acid decreases enzyme activity by changing the ideal structure of the substrate pocket^14,25^. Therefore, it possible that the rs30187 T allele (Arg528Lys), which epistatically interacts with HLA-B*51, leads to a greater ERAP1 enzymatic activity involving high efficiency peptide trimming, which may contribute to the BD susceptibility.

In summary, this study in an Iranian population showed an interaction between *ERAP1* rs30187 recessive genotype and HLA-B*51. It is suggested that HLA-B*51 and *ERAP1* variants may interact with eachother and confere a modified risk for BD development, however, *ERAP1* variants which interact with HLA-B*51 may differ among various populations and diverse disease phenotypes. The association of rs30187 with articular and cardiac manifestations was also identified in BD patients. Taken together, these findings provide strong but still inconclusive evidence that targeting this molecular interaction might be beneficial in certain subgroup of patients harboring rs30187 recessive genotype.

## Data Availability

Data will be available upon request.
